# microRNAs as New Biomolecular Markers to Estimate Time since Death: A Systematic Review

**DOI:** 10.3390/ijms25179207

**Published:** 2024-08-24

**Authors:** Vincenzo Cianci, Cristina Mondello, Daniela Sapienza, Maria Cristina Guerrera, Alessio Cianci, Annalisa Cracò, Francesco Luppino, Vittorio Gioffrè, Patrizia Gualniera, Alessio Asmundo, Antonino Germanà

**Affiliations:** 1 Department of Biomedical and Dental Sciences and Morphofunctional Imaging, Section of Legal Medicine, University of Messina, Via Consolare Valeria, 1, 98125 Messina, Italy; mondelloc@unime.it (C.M.); patrizia.gualniera@unime.it (P.G.); alessio.asmundo@unime.it (A.A.); 2 Zebrafish Neuromorphology Lab, Department of Veterinary Sciences, University of Messina, Via Palatucci snc, 98168 Messina, Italy; mariacristina.guerrera@unime.it (M.C.G.); agermana@unime.it (A.G.); 3 Department of Cardiovascular Medicine, Fondazione Policlinico Universitario A. Gemelli-IRCCS, Largo A. Gemelli 8, 00168 Rome, Italy; alessiocianci.1998@gmail.com; 4 Department of Biomedical Sciences and Morphological and Functional Imaging, Diagnostic and Interventional Radiology Unit, University Hospital Messina, 98125 Messina, Italy; annalisacraco@hotmail.it; 5 Department of Clinical and Experimental Medicine, University of Messina, 98125 Messina, Italy; fralup97@gmail.com; 6 Department of Otorhinolaryngology-Head and Neck Surgery, IRCCS San Raffaele, Vita-Salute San Raffaele University, Via Olgettina 60, 20132 Milan, Italy; vittoriogioffre@outlook.it

**Keywords:** PMI estimation, post-mortem interval estimation, RNA degradation, microRNA degradation, miRNA degradation, forensic molecular pathology, forensic pathology

## Abstract

Estimating the post-mortem interval is still one of the most complex challenges in forensics. In fact, the main tools currently used are burdened by numerous limitations, which sometimes allow the time of death to be placed only within too large time intervals. In recent years, researchers have tried to identify new tools to try to narrow down the interval within which to place the time of death; among these, the analysis of microRNAs seems to be promising. An evidence-based systematic review of the literature has been conducted to evaluate the state of the art of knowledge, focusing on the potential correlation between miRNA degradation and PMI estimation. The research has been performed using the electronic databases PubMed, Scopus, and WOS. The results allowed us to highlight the usefulness of miRNAs both as markers for PMI estimation and for normalization, especially due to their stability. In fact, some miRNAs remain particularly stable for long periods and in different tissues, while others degrade faster. Furthermore, there are numerous factors capable of influencing the behavior of these molecules, among which the type of tissue, the cause of death, and the circadian rhythm appear to be the most relevant. Despite the promising results of the few articles present in the literature, because of the numerous limitations they are burdened by, further research is still necessary to achieve more solid and shareable results.

## 1. Introduction

The “post-mortem interval” (PMI) refers to the time occurring between an individual’s death and its body finding [1,2]. Although accurately estimating the PMI is critical in the operations of forensic pathologists, there are currently no procedures that allow for its perfect calculation [3]. In fact, to date, only relatively small time spans in which to place the time since death can be estimated [1]. In particular, depending on the circumstances, the post-mortem delay might be estimated in hours, days, or even years. The postmortem interval has typically been divided into four stages: immediate, early, intermediate, and late [4,5,6]. Despite that, there are no universally accepted criteria that allow each period to be differentiated within well-defined time frames [1]. For instance, according to some authors, the immediate post-mortem phase occurs within two or three hours after death [4]. During this period, no significant postmortem phenomena are detectable [4].

The early post-mortem (ePMI) period is considered the most crucial time period for estimating post-mortem intervals, since it is during this period that cadaveric phenomena occur. The ePMI ranges from 3 to 72 h after death [5]. Therefore, this is the period in which the estimation of time since death acquires the greatest importance [5]. The most widely used and well-known methods to estimate the ePMI are represented by the evaluation of rigor mortis with reference to the maneuver of reversal of cadaveric rigidity, the variation of both environmental and cadaveric temperature analyzed through the thermometric method (Henssge nomogram), and the evaluation of the main hypostases’ characteristics [6]. Despite this, it is well recognized that the usefulness of these methods in calculating PMI is affected by both intrinsic and extrinsic confounding variables, which might influence their reliability, particularly when post-mortem intervals reach 24–48 h [3,7]. These issues became evident from the start of using these techniques, necessitating the development of many adjustment factors. It was necessary to use two different Henssge nomograms to reduce the percentage of error related to the environmental temperature value; therefore, a cut-off value of 23 °C relating to the environmental temperature was introduced [8]. Despite this, it is known that the nomogram is only useful between 24 and 48 h after death, with an error variability that can surpass 7 h [8]. Other external factors typically include the subject’s clothes and insulation, as well as the body’s storage after death [9]. Factors that minimize the rate of post-mortem modifications include freezing ambient conditions, sparsely dressed or naked subjects, and the body being stored in a refrigerator after death [9,10]. The most important intrinsic elements capable of accelerating or decelerating postmortem alterations are body mass and surface area [10].

Given the ever-increasing necessity to minimize the time gap within which to record the time since death, the scientific community has attempted to develop new methods that allow these limitations to be circumvented, resulting in a large increase in forensic research. In fact, the relevant literature now includes investigations on the use of forensic entomology, microbiome variation, metabolomics analysis, and protein, DNA, and RNA degradation analysis [11,12,13,14,15,16].

In recent years, advances in molecular techniques used to examine the degradation of nucleic acids and proteins have enabled the evaluation of new approaches for calculating the post-mortem interval [12]. The degradation of deoxyribonucleic acid (DNA) and ribonucleic acid (RNA) has received special attention [11,12].

In general, DNA and RNA molecules have been shown to degrade over time, despite being influenced by a variety of intrinsic and extrinsic factors, such as environmental temperature [11,12]. Some studies have documented a variable degradation time of the same RNA within the same tissue when held at different temperatures [12]. Similar considerations can be made when evaluating the degradation times of the same molecules (RNA, DNA, proteins) in various tissues (e.g., heart vs. skeletal muscle): even when held at the same temperature, variable degradation times for the same molecule have been reported [17]. This suggests that internal variables can impact the degradation time of a single molecule [18]. Similar results have been obtained by assessing RNA molecules: it appears that the rate of degradation is directly proportional to the body mass index (BMI), increasing as the BMI increases [9,10]. Despite the aforementioned limitations, it is important to note that the employment of these methods, especially when combined with the “common” ones, appears to be highly promising for achieving a more exact assessment of the PMI [17].

RNAs are commonly divided into two large families: coding RNA and non-coding RNA; miRNAs belong to the second group [19]. miRNAs are usually made up of 20–24 nucleotides and are involved in the regulation of gene expression. In particular, miRNAs intervene in gene post-transcriptional regulation [20]. Their task is usually to silence their target genes after binding to complementary sequences of the correspondent mRNAs [20]. Therefore, miRNAs play a pivotal role in several biological processes, including development, differentiation, proliferation, and apoptosis [21]. Because of the role played in apoptosis, some miRNAs could also increase in the post-mortem era, stimulating positive cellular degradation phenomena [21].

To use these molecules as PMI indicators, it is also necessary to be able to quantify their changes as precisely as possible within well-defined time intervals [22]. For this reason, quantitative real-time polymerase chain reaction (qRT-PCR) is the method of choice [22].

However, it is known that there are some conditions, such as tissue sampling, that can affect the real concentrations of RNAs, resulting in changes in gene expression and then inaccurate data interpretation [23]. For these reasons, proper data standardization is considered of great importance [24]. As a result, when conducting research on RNA degradation, reference genes must be used for normalization [24]. Reference genes are internal reaction controls whose sequences differ from those of the target. To be regarded as a reliable reference, a gene must meet stringent criteria [25]. Among these, the most important is thought to be its expression level, which must remain stable in experimental settings [25]. Currently, GAPDH and β-actin are widely used and suggested as endogenous reference genes [26].

The purpose of this review is to conduct a systematic analysis of the most recent and relevant literature on the potential utility of evaluating the degradation of diverse microRNA types as a new tool for a more exact assessment of PMI.

## 2. Methods

### 2.1. Search Strategy

An evidence-based systematic review of the literature has been conducted according to the PRISMA guidelines, evaluating the main research focusing on a new frontier for post-mortem interval estimation, represented by the evaluation of microRNA degradation.

The following search queries were used: (i) microRNA degradation and PMI estimation; (ii) microRNA degradation and postmortem interval estimation; (iii) miRNA degradation and postmortem interval estimation; (iv) miRNA degradation and postmortem interval estimation; (v) miRNA degradation and time since death and forensic pathology (vi) miRNA degradation and early PMI estimation (vii) ((miRNA degradation) AND (PMI estimation)) OR (post-mortem interval estimation). The last date of search was 26 June 2024.

### 2.2. Data Extraction

**Figure 1:**
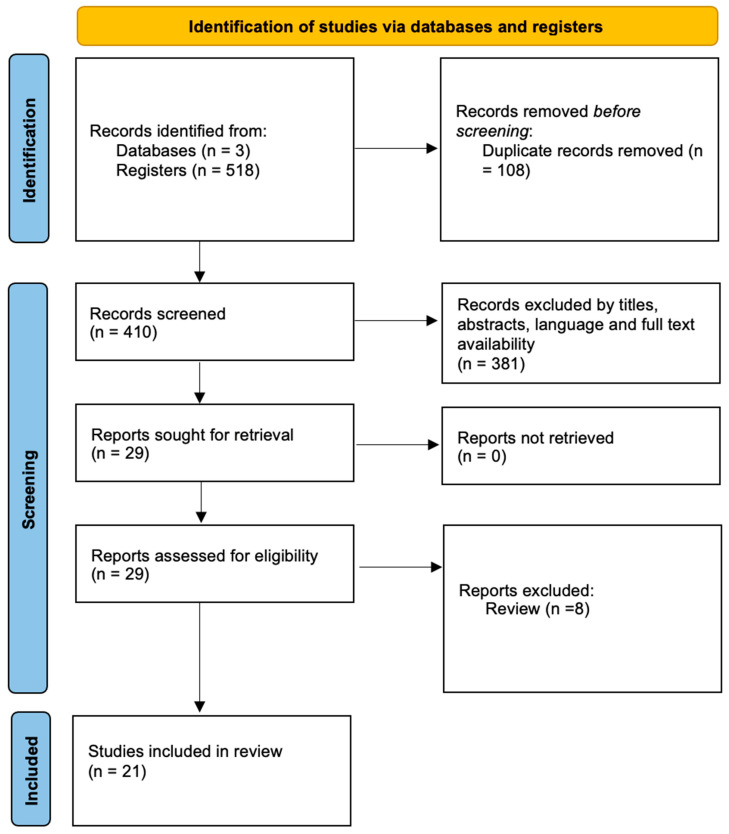
PRISMA 2020 flowchart.

The electronic databases PubMed, Scopus, and WOS have been used to search for articles to enlist in the review. The search has then been extended by checking the reference lists of the articles principally eligible for inclusion. Specific inclusion criteria, such as the full text availability, the English language, and year of publication before 2013, were used for the pre-selection of the articles. Duplicates have been removed. Some articles have been removed by evaluating titles and abstracts that were not consistent with the systematic review (Figure 1).

According to the 2020 PRISMA guidelines (supplementary materials, the following supporting information can be downloaded at: https://www.mdpi.com/article/10.3390/ijms25179207/s1), the enrolled articles have been separately evaluated by three of the authors. Data extraction has been performed by two investigators; the data were then verified by two more authors. The systematic review was not registered with a public registry.

## 3. Results

In an attempt to overcome the limitations that characterize the conventional methods used for PMI estimation, in recent years, new tools have been proposed, including the evaluation of the degradation times of a particular category of RNA, the microRNAs. In Figure 1, the main results of the enrolled articles are summarized. In particular, the selection process led to the enrollment of 21 articles, including original articles, short communications, case reports, and case series.

### 3.1. Risk of Bias

The systematic review has some strengths, such as the detailed description of the enrolled articles and the strict criteria used for the selection process. Despite that, it must be highlighted that the review includes studies that have been published in a time frame of 11 years, as the authors focused on the most recent articles. Therefore, articles before 2013 were excluded. Furthermore, the obtained data could have an intrinsic risk of bias due to the different analysis tools that have been used by each research group.

### 3.2. miRNA and PMI Estimation

#### 3.2.1. miRNA Expression Patterns Assessed within PMI of 72 h

Odriozola A. et al. [27] conducted an analysis on the degradation times of miR-34c, miR-541, miR-888, miR-484, miR-142-5p, and miR-222, sampled from the vitreous humor of a population of 34 corpses who underwent autopsy. Due to its particular stability, miR-222 was chosen for normalization. All the microRNAs studied showed particular stability, at least within the first 24 h after death. Despite this, the objective of this study was to evaluate whether the period of the day in which death occurred could be associated with a variation in the concentration of microRNAs; indeed, the results allowed to highlight an upregulation of miR-142-5p and miR-541 if deaths occurred at nighttime. Conversely, miR-484 was always shown to be downregulated, as was miR-34c, which decreased as the PMI increased. Given the high stability of microRNAs, the authors concluded that their degradation does not correlate with PMI within 24 h.

Sharma et al. [28] hypothesized that the degradation time of some RNAs may be influenced by the circadian rhythm. A population of nine rats was enrolled, all kept at an environmental temperature of 25 °C, and subsequently divided into three groups based on the time in which they were sacrificed (at 4 a.m., 8 p.m., and 12 p.m.). Sampling was performed on the kidneys, liver, spleen, pancreas, brain, heart, and lungs for a total of 72 h. The selected RNAs were AATF mRNA and miR-2909. The authors described a statistically significant correlation between the variation in AATF mRNA and miR-2909 levels and the circadian rhythm. In particular, AATF mRNA levels remained stable up to 24 h if mice were sacrificed at 8 p.m. and up to 12 h if sacrificed at 12 p.m., whereas miR-2909 levels remained stable up to 48 h if mice were sacrificed at 8 p.m. and up to 12 h if sacrificed at 12 p.m.

Lu Y.H. et al. [29,30] focused on the importance of building predictive mathematical models for PMI estimation. In both studies, the degradation patterns of miRNA-9, miRNA-125b, β-actin, GAPDH, RPS29, 18S rRNA, 5S rRNA, and U6 snRNA were analyzed. 5S rRNA, miRNA-9, and miRNA-125b were instead used for normalization. Initially, the authors evaluated the degradation times of the aforementioned RNAs on an animal model: a population of 222 rats was enrolled and randomized into five sub-groups, providing a control group (PMI 0 h) and four further groups. The samples were taken from the brain tissue within a maximum observation period of 24 h (1, 2, 4, 6, 8, 10, 12, 24). The groups were stored at different temperatures, ranging from 5 to 35 °C. Conversely, in their second research, the same RNAs were studied in the brain tissue of 12 corpses to establish a human mathematical model. Cadavers with known PMI ranging from 4.3 to 22.5 h were enrolled, but it was not possible to conduct the study at controlled temperatures. β-actin and GAPDH mRNA showed progressive degradation as PMI increased, while miRNA-9 and miRNA-125b showed high stability during the first 24 h, proving to be suitable as internal reference markers in both rats and human brain tissue. After validation, the average error rates in rat brain tissue using β-actin and GAPDH were 14.1% and 22.2%, respectively, while they increased to 24.6% and 41.0% in human brain tissue.

Ibraim S.F. et al. [31] enrolled a population of 18 albino rats to analyze the degradation times of miR-205 and miR-21 on the skin matrix. The time interval covered by the study was between 0 and 48 h. Although it was possible to identify a strong increase in the concentrations of both miRNAs at 24 h and a strong decrease at 48 h, the statistical analysis did not allow for the identification of statistically significant correlations between the variation in their concentrations and the PMI.

Han L. et al. [32] analyzed the variations in miR-3185 levels in the cardiac tissue of 51 autopsied cadavers. The enrolled sample was divided into five groups in relation to the cause of death: the first group consisted of 14 deaths due to mechanical asphyxia, the second 14 cases of craniocerebral injury, the third 14 cases of hemorrhagic shock, the fourth five cases of sudden cardiac death, and the last four cases of poisoning. The sample was normalized with β-actin mRNA. All enrolled cadavers were subjected to autopsy and subsequent sampling within 1.5 to 30.5 h of death, with temperatures varying from 5 to 40 °C. The results obtained made it possible to highlight a marked increase in miR-3185 in the group of subjects who died from mechanical asphyxia compared with other modalities. No correlations were highlighted between the variation in the quantities of miR-3185 and the PMI.

The PMI-related variations of miR-23-3p, miR-381-3p, and miR-114-3p have been analyzed by Martinez-Rivera V. et al. [33]. The PMIs of interest were established at 0, 3, 6, 12, and 24 h. The study was conducted on the skeletal muscle of 25 rats, maintained at a controlled temperature of 25 °C, and randomized into five groups based on PMI. The first group, at 0 h PMI, was considered the control group. The gene expression of each miRNA has been expressed as a fold change (FC). The analysis of miR-381-3p allowed us to identify a “J-shape curve” trend; in fact, after an initial downregulation during the first 3 h, a constant upregulation was found for up to 24 h. Instead, for miR-23b-3p, a progressive reduction was observed up to 24 h. Finally, for miR-114-3p, a progressive reduction was observed from 0 to 6 h. The expression variations of the miR-381-3p target gene, the EPC1 gene, were found to be downregulated at 3 and 12 h PMI, but not at 6 and 24 h. Statistically significant associations between the variation of some miRNAs and PMI were identified: 0, 3, 12, 24 h, but not at 6 h for miR-381-3p or 24 h for miR-23b-3p, as it was found to be significantly reduced if compared with the control group (0 h PMI). No statistically significant associations were identified for miR-144-3p.

Derakhshanfar A. et al. [34] analyzed the degradation pattern of miR-122 on both the liver and kidney of a population of 10 rats, aiming to evaluate the possible influence of the suppression methods on miRNA expression. Therefore, rats were randomized into two groups. The first group was euthanized via exposure to a rising concentration of CO_2_, and the second one via a high dose of intramuscular ketamine injection. The samples were taken at 2, 4, 6, 8, 10, 12, 24, and 48 h, and the samples were stored at 25 °C. miR-16, miR-221, Let-7a, and U6 snRNA were selected for normalization. miR-122 was upregulated in the drug-treated group at 4, 10, and 24 h. On the contrary, comparing at 6, 8, and 48 h the miR-122 levels of the second group to the first, they were shown to be downregulated. The authors concluded that the mode of sample suppression during the experiments can play an important role in the variation of miRNA levels; therefore, further studies are needed to identify the best one.

Guardado-Estrada M. et al. [35] conducted a study on miRNA expression variation in rat skeletal muscle within 24 h of death (early PMI). Nina rats were enrolled, kept at a temperature of 22 °C, and subsequently randomized into two groups based on PMI: four constituted the control group (0 h PMI) and five the other group (24 h PMI). A total of 1.218 rat miRNAs were analyzed using the Affymetrix GeneChip miRNA 4.0 array probes of mature rats’ miRNAs. A total of 156 miRNAs underwent changes within 24 h, resulting in 84 being downregulated and 72 being upregulated. Among these, miR-139-5p was found to be the most significantly downregulated, while rno-miR-92b-5p was the most significantly upregulated. In the study, interactions between miRNAs and mRNAs were also observed: interactions with 130 different mRNAs were found for rno-miR-125b-5p/rno-miR-351-5p, and 120 for rno-miR-138-5p. The gene expression of TGFBR2, SIRT1, and BMF mRNAs, which are respectively the targets of rno-miR-291a-3p, rno-miR-125b-5p/rno-miR-351-5p, and rno-miR-138-5p, was subsequently analyzed with qRT-PCR. These mRNAs were found to take part in postmortem autophagy, cell cycle regulation, and the low oxygen response. SIRT1 mRNA was found to be downregulated at 24 h, while TGFBR2 and BMF mRNAs were upregulated. Statistically significant associations between mRNA degradation time and PMI were found for SIRT1 and TGFBR2, but not for BMF.

The main data of the selected articles are summarized in Table 1.

#### 3.2.2. miRNA Expression Patterns Assessed within PMI of 7 Days

Wen-Can L. et al. [36] analyzed the degradation pattern of some miRNAs and rRNAs over a time interval of 7 days. The enrolled sample consisted of six rats killed by suffocation and stored at 25 °C. The analysis was conducted on the heart, sampled at regular intervals (1, 3, 6, 12, 15, 18, 21, 24, 36, 48, 72, 96, 120, 144, and 168 h, up to 7 days). The selected RNAs were represented by miR-1, miR-143, miR-208, and 18S rRNA. The analysis performed allowed the following results to be obtained: 18S-rRNA showed a gradual increase in the first 96 h and then progressively decreased in the subsequent time intervals. On the contrary, miR-1 remained stable up to 168 h and was therefore selected as an endogenous reference gene to normalize 18S-rRNA. The linear regression analysis of 18S-rRNA allowed us to obtain better values than the regression of 18S-rRNA/miR-1; despite this, the authors proposed the adoption of the latter, as normalization reduces the probability of error in estimating the PMI. The authors concluded that miR-1 can be used as a reference gene in cardiac tissue.

Pan H. et al. [37] analyzed the degradation time of GAPDH and β-actin mRNA, miR-203, 18S rRNA, and 5S rRNA on the skin samples of 18 rats. The enrolled population was randomized into three groups, respectively, and stored at 4, 15, and 35 °C. The selected time interval ranged from 0 to 120 h after death. Both miR-203 and 5S rRNA proved to be the ones with greater stability and, therefore, are the most suitable as internal controls. At 4 and 15 °C, β-actin and GAPDH mRNA had a linear relationship, while at 35 °C, they formed a sigmoid curve. At 15 and 35 °C, 18S rRNA had a partial linear association with PMI. GAPDH and β-actin mRNA analyzed on skin samples were considered an appropriate matrix for studying RNA degradation, while miR-203 can be used as a reliable internal control.

Ma J. et al. [38] chose Let-7a, 18S rRNA, miR-125b, miR-9, GAPDH, RPS29, 5S, β-actin, and U6 snRNA as RNAs of interest, analyzing their degradation patterns on the brain tissue of 270 rats killed by cervical dislocation. The rats were divided into five groups: the first group was used as a control, and sampling was performed immediately after the sacrifice, at time 0. The other groups were further divided according to both PMI (1, 3, 6, 12, 24, 36, 48, 72, 96, 120, and 144 h) and temperatures at which the sample was stored (4, 15, 25, 35 °C). For normalization, one more group made up of 36 rats was added: samples were taken at 10, 30, 50, and 100 h and maintained at three different temperatures (10, 20, and 30 °C). The results showed the high tissue specificity and stability of miR-9 and miR-125b, making them a useful tool for estimating PMI up to 144 h. Conversely, because of the rapid degradation time of β-actin, it was used to produce and validate a mathematical model showing a low error percentage, ranging from 30% (30 h at 20 °C) to 43% (10 h at 30 °C). The authors concluded that brain-specific miR-9 and miR-125b can be considered excellent endogenous controls, as they remain stable up to 144 h after death at temperatures between 10 and 35 °C.

Lv Y. et al. [39] analyzed the degradation patterns of miR-195, miR-200c, 5S, U6, and RPS29 in lung tissue and miR-1, miR-206, 5S, and RPS29 in skeletal muscle. The research was conducted by enrolling 216 rats, randomized into three groups. The first group was further divided into three sub-groups according to three different temperature values (10 ± 1 °C, 20 ± 1 °C, and 30 ± 1 °C), and the samples were taken at 0, 1, 3, 6, 12, 24, 36, 48, 72, 96, 120, and 144 h after death. To validate the model, a second group of 15 rats was randomized into 5 sub-groups according to the storage temperature (10, 15, 20, 25, 30 °C), and samples were taken at 10, 60, and 110 h after death. Furthermore, to evaluate the degradation time of the same RNAs on humans, the 12 cadavers that died between 7 and 73 h were analyzed; in this setting, temperature was not recorded. The upper lobes of the left lung and the quadriceps femoris of the right leg were selected as sampling areas. △Ct values of both ACTB and GAPDH increased faster in lung than in muscle tissue at the same temperature, both proving valid for estimating the PMI. miR-1 showed high stability, making it useful for normalization. It has been proven that mathematical models could decrease the error rate in PMI estimation, resulting in an error rate of 7.4% for rats and 12.5% for humans.

Another study by Lv Y. et al. [40] analyzed miR-1, miR-133a, miR-122, miR-9, and miR-125b degradation patterns on the myocardial tissue (apex cordis), the right lobe of the liver, and the frontal cortex of the brain of 13 cadavers, with known PMI varying between 6 and 71 h. Samples were stored at 4, 15, 25, and 35 °C, while β-actin, GAPDH, and RPS29 mRNA were used as reference RNA for normalization. miR-122 appears to degrade along the PMI in the liver, especially at high temperatures. On the other hand, miR-133a and miR-1 were highly stable in the myocardium for more than five days, not being influenced by temperature variations. miR-1 and miR-133a were then considered suitable as reference genes in the heart tissue; conversely, miR-122 cannot be used as a reference gene in the liver due to its instability and rapid degradation. The authors also performed an estimate of the error, expressed as “mean estimated error”, which was higher on the human sample (5.06) compared with that taken from rats (2.98).

Kim S-Y. et al. [41] conducted an innovative study evaluating the degradation times of four miRNAs in the blood matrix. A population of 28 cadavers (17 male, 11 female) of subjects who died from various pathologies, all with known PMI varying between 16 and 86 h, was enrolled. Two cardiac-specific miRNAs (miR-208b and miR-1) and two non-cardiac-specific miRNAs (Let-7e and miR-16) were selected. Blood samples were performed on peripheral blood during the autopsy; venous blood was collected from the external iliac vein, the inferior vena cava, and the coronary sinus. The enrolled sample was subsequently divided based on two criteria: cardiac deaths vs. non-cardiac deaths, and subjects who underwent cardiopulmonary resuscitation (CPR) vs. non-CPR. In all groups, the degradation times of the selected miRNAs were analyzed. The obtained results allowed us to observe a variation in the quantity of cardio-specific miRNAs (miR-208b and miR-1) related to the sampling site, while no differences for the non-cardio-specific ones (Let-7e and miR-16) were detected. No statistically significant differences comparing cardiac vs. non-cardiac deaths or CPR vs. non-CPR were detected. No statistically significant results for a possible correlation with the PMI were obtained. Therefore, the analysis of the aforementioned miRNAs in peripheral venous blood negatively correlates with the PMI.

The main data of the enrolled articles are summarized in Table 2.

#### 3.2.3. miRNA Expression Patterns Assessed within PMI over 7 Days

Lv Y. et al. [42] enrolled a population of 18 rats, subsequently randomized into two groups. The first group was further divided into two other groups, stored at 4 and 25 °C, respectively. For each group, splenic samples were taken at 0, 1, 3, 6, 12, 24, 36, 48, 72, 96, 120, and 144 h, and at 0, 12, 24, 36, 48, 72, 96, 120, 144, 168, 192, 216, 240, 264, 288, and 312 h after death, respectively. A third group of six rats was added to validate the mathematical model: two subgroups of three rats each, maintained at the same temperature, underwent sampling at 0, 5, 55, and 105 h, and at 0, 20, 100, 180, and 260 h after death. After validation, miR-125b and miR-143 microRNAs were identified as endogenous control markers, as they were less affected by both temperature and PMI. Conversely, at 25 °C, β-actin1 and GAPDH1 showed a slight cubic curve, while β-actin2 and GAPDH2 showed a rapid decrease; thus, β-actin2 and GAPDH2 were considered useful in determining early PMI. In the study, the percentage error related to the temperature parameter was also calculated: this was less than 10% for temperatures of 25 °C and less than 20% for the sample kept at 4 °C.

Tu C. et al. [43] analyzed the degradation times of different RNAs in a mouse model, enrolling a population of 45 healthy adult male mice killed by cervical dislocation. Mice were subsequently randomized into nine groups based on the PMI (0, 1, 2, 3, 4, 5, 6, 7, 8 days), each group made up of five mice; the first group (PMI = 0 d) was used as a control. The entire enrolled population was maintained at a temperature of 25 °C. Samples of cardiac, hepatic, and skeletal muscle tissue were then collected from each mouse, and the degradation times of 11 candidate genes were analyzed: miR-133a, miR-122, GAPDH, Rps18, β-actin, 5S, 18S, U6, circ-AFF1, LC-Ogdh, and LC-LRP6. The obtained results made it possible to identify the greater stability of miRNA and circRNA when compared with other RNAs, making them more suitable for this purpose. Differences in the expression of the same RNAs in the three different tissues analyzed were highlighted: both miR-133a and miR-122 proved to be equally stable on cardiac tissue, while miR-133a showed greater stability in skeletal muscle and less in the liver, opposite behavior for miR-122, which was more stable in liver and less in skeletal muscle. The authors also highlighted that circRNAs are more stable than miRNAs in the liver.

To build a mathematical model for evaluating the relationship between the postmortem interval and the expression levels of target biomarkers, Tu C. et al. [44] conducted another study on a population and tissues identical to the previous one [43]. In this study, 15 healthy adult male mice—killed by cervical dislocation—were enrolled and subsequently randomized on the basis of PMI (0, 1.5, 3.5, 5.5, and 7.5 days) into five groups, each composed of three mice. As in the previous study, the samples were taken from the heart, liver, and skeletal muscle and then stored at 25 °C, with the first group serving as the control. The same biomarkers were chosen: GAPDH, RPS18, U6, and β-actin as candidate target biomarkers; miR-122, miR-133a, 18S, LC-Ogdh, and circ-AFF1 as reference genes for the three tissue types. The model obtained allowed us to confirm that RNA, and in particular miRNAs, can be used to estimate the post-mortem interval with a certain accuracy. In particular, the low estimated error on the validated samples was equal to 0.62 d. Furthermore, if the data obtained from the three analyzed tissues were integrated, the error rate would be further reduced to 0.5 days.

Alshehhi S. et al. [45] analyzed the degradation patterns of miR205 and STATH mRNA on saliva and PRM1 and PRM2 mRNAs, miR891a and miR10b, on semen of 19 voluntary living donors. The observation period lasted one year, and the following time intervals for sampling were defined: 0, 7, 14, 28, 90, 180, 270, and 360 days. The samples were maintained at room temperature. ACTB, 18S, and U6 were chosen as reference genes for normalization. A high stability of miR891a and miR205 up to 360 days was described, while miR10b remained stable only within the first 14 days. STATH mRNA remained stable for up to 28 days, and PRM1 and PRM2 mRNA remained stable for up to 90 days. The authors concluded for the greater stability of miRNA miR891a and miR205.

Na J-Y. et al. [46] finally conducted a study evaluating the degradation times of two miRNAs, Let-7e and miR-16, for the estimation of the “long” PMI. The study was conducted by extracting the patella from 71 different cadavers during an autopsy. The sample was subsequently randomized into four groups based on the different PMIs, globally ranging from 1 day to 2 years. The first group consisted of 37 patellae, with a known PMI of less than 1 month. The second group consisted of 18 patellae with PMI between 1 and 3 months; the third group included 11 patellae with PMI between 3 and 6 months; and the last group consisted of five patellae with PMI greater than 6 months. The expression levels of miRNAs were normalized with Ce_miR-39_1. The results provided allowed us to evaluate the rapid degradation of miRNAs in the first month, resulting in significantly different levels of both Let-7e and miR-16 between group A and the other three groups. The authors concluded that the study of miRNAs in bone tissue could be a promising tool for estimating PMI, although statistical significance was reached within the first month.

Singh P. et al. [47] analyzed the degradation timing of three different miRNAs: miRNA-195, miRNA-206, and miRNA-378. miRNA-1, which is known to be stable up to 196 h after death, was used as an endogenous control for normalization. miRNA-195 and miRNA-206 have already been studied and have shown evidence, while miRNA-378 is being analyzed for the first time. The study was conducted on left ventricular wall samples from 20 hearts of subjects who died in road accidents, aged between 18 and 32. For the selection of this sample, two exclusion criteria were adopted: unknown PMI and the possibility of performing an autopsy no later than 12 hours after death. The analysis conducted allowed us to identify a statistical significance between the degradation time of these three miRNAs and the PMI. More specifically, a statistically significant reduction in the concentrations of each miRNA was observed when compared at 24 and 196 h, outlining their usefulness in estimating PMI in this time interval.

The main data of the selected articles are summarized in Table 3.

## 4. Discussion

### 4.1. Main Evidence on microRNAs in PMI Estimation

Currently, one of the most challenging tasks of forensic pathology is trying to estimate the postmortem interval as precisely as possible [48]. For this reason, the scientific community is trying to identify new tools that are not burdened by the most common limitations of conventional methods [49]. From this perspective, even in the forensic field, research has focused on molecular diagnostics in the attempt to identify target molecules that can be useful for this purpose [49]. Undoubtedly, the analysis of RNA degradation times is gaining increasing interest, especially as regards microRNAs [50].

In this context, miRNAs have emerged as valuable biomarkers for PMI estimation, especially because of their stability and distinct degradation patterns [51]. Among them, miR-133a, miR-125b, and miR-1 have been identified as reliable reference genes as they show consistent degradation rates, making them suitable for data normalization [28,29,30,36,38,42,47]. Anyway, to further reduce the error percentage associated with erroneous normalization, the geNorm and Normfinder algorithms created a set of reference genes, specific for each tissue, that may be used to improve the accuracy of the research [26].

When performing this type of research, the introduction of mathematical models is another important factor in limiting errors caused by inaccurate data understanding and interpretation [51]. It is now known that developing mathematical models can reduce the error rate when complex data are analyzed [51]. As a result, if used, mathematical models would increase both precise predictions and accurate quantification of correlations between several variables, enhancing the interpretation of wide datasets and reducing error rates [52]. These data have been confirmed in some research, where the use of mathematical models has made it possible to obtain quantification and a significant reduction in the error percentage [36,42]. Sampaio-Silva et al. [53] validated a mathematical model for PMI estimation on rats’ skeletal muscle and several other organs, obtaining a 95% confidence interval. The authors found ACTB, GAPDH, Ppia, and Srp72 genes to be related to PMI if their quantitative evaluation is performed on skeletal muscle.

As previously said, although some mRNAs, such as β-actin and GAPDH, are considered among the best markers for data standardization, it is known that they tend to degrade in relatively narrow periods, reducing their reliability as PMI indicators [51]. For this reason, research has moved towards the identification of more stable markers, such as microRNA [54]. There are several reasons why microRNAs present greater stability than other classes of the same molecule, among which their small size, their association with protein complexes (such as RISC), or even their high “affinity binding” are considered the most relevant [55].

In the literature, several articles have highlighted the usefulness of some miRNAs for normalization; among these, miR-222 [27,43,44], miR-1 [36,39,47], miR-125b [29,30,38,42], and miR-9 [28,38,39] were those with greater reliability. However, miRNA stability can vary depending on the tissue type examined [56]. Therefore, it is necessary to confirm that miRNA stability remains unchanged in other tissues different from those already studied. If not, its reliability would be reduced [56]. For example, Lv Y. et al. [40] described a particular stability of miR-1 and miR-133a on cardiac tissue, defining them suitable as reference genes, while a too rapid degradation of miR-122 in liver was observed. In contrast, Tu C. [43] described the greater stability of miR-122 in the liver compared with skeletal muscle. Tu C. [43] also confirmed the high stability of miR-133a in the heart, while a more rapid degradation was documented in liver tissue.

Another factor that seems to influence the stability of microRNA is represented by the circadian rhythm, as demonstrated in “in vivo” studies [57]. Therefore, some authors evaluated whether the circadian rhythm, understood as the time of day in which death occurred, could determine a variation in microRNA concentration [27,28]. Odriozola A. et al. [27] documented an upregulation of miR-142-5p and miR-541 if deaths occurred at nighttime. Conversely, miR-484 and miR-34c always remained downregulated. Sharma et al. [28] instead described the behavior of miR-2909 as influenced by the circadian rhythm: it remained stable up to 48 h if rats were sacrificed at 8:00 p.m., while its stability was reduced to 12 h if rats were sacrificed at 12:00 p.m. In the same study, a similar behavior of AATF mRNA was also documented: it remained stable up to 24 h if sacrificed at 8:00 p.m. and up to 12 h if sacrificed at 12:00 p.m. Therefore, the stability of some miRNAs appears to be influenced by the circadian rhythm, although there are still numerous studies that seek to understand, even in living beings, the mechanisms by which this occurs [58]. More specifically, circadian rhythms are governed by a set of core clock genes that drive the rhythmic expression of a large number of genes [58]. These rhythms ensure that physiological processes occur at optimal times during the day and night [58]. Among these, there are those intervening in the processes of programmed cell death, also occurring in postmortem settings [58].

Some studies showed that specific miRNAs, such as miR-21, miR-34a, and miR-125b, which are known to play a key role in regulating apoptosis, would appear to be influenced by the circadian rhythm, also influencing cell apoptosis at different hours [59,60,61].

miR-34a is regulated by tumor suppressor protein (p53) and promotes apoptosis by targeting multiple genes, such as BCL2 (B-cell lymphoma 2) and SIRT1 (Sirtuin 1) [59]. In particular, p53 activation determines the increase of miR-34a, which is in turn responsible for the activation of cell death processes. Induction of miR-34a in response to p53 activation leads to increased apoptotic cell death, making it a critical player in the p53-mediated apoptotic pathway [60]. On the other hand, miR-125b has a dual role in apoptosis, acting as an oncogene or tumor suppressor. For example, in several tumors, miR-125b can block apoptosis by inhibiting pro-apoptotic genes such as BAX (Bcl-2-associated protein X) and PUMA (upregulated modulator of apoptosis p53) [61]. In post-mortem settings, miR-125b levels could help to determine the time since death; its levels are usually increased, reflecting both an activation of the apoptosis pathways and a response to hypoxia because of the postmortem cessation of the circulation [60,61].

Therefore, the time of death can significantly affect the quantity of miRNA levels detected in post-mortem tissues, which is crucial for its correct assessment. In fact, the mere quantification of miRNA levels, if not appropriately standardized, could lead to the acquisition of discordant results [62]. Despite this, in the forensic field, evidence is still very limited, and therefore numerous studies are needed to quantify and standardize the variations of these biomolecules so as to further reduce the percentage of error in estimating the time of death by means of their use [27,28,62].

In addition to the analyzed matrix and the circadian rhythm, another factor capable of modifying the expression of miRNAs could be represented by the cause of death [32,34]. Han L. et al. [32] analyzed the variations in miR-3185 levels in the cardiac tissue of 51 autopsied cadavers, deceased from craniocerebral injury, hemorrhagic shock, sudden cardiac death, poisoning, and mechanical asphyxia. The authors detected RNA downregulation in all cadavers, except those of subjects who died of mechanical asphyxia, where it resulted in upregulation. miR-3185 regulates the expression of hypoxia-inducible factors (HIFs) or other hypoxia-related genes, influencing the stability and translation of mRNAs encoding proteins involved in angiogenesis, metabolism, and cell survival; for this reason, it could be upregulated in asphyxia deaths [63]. Derakhshanfar A. et al. [34] obtained similar results evaluating the variations of miR-122 on both the liver and kidney of 10 rats suppressed with an arising concentration of CO_2_ or via a high dose of intramuscular ketamine injection. miR-122 was upregulated in rats sacrificed by CO_2_.

Furthermore, although miRNAs are considered excellent markers for normalization thanks to their stability, some authors have proposed the analysis of their degradation times as an indicator of the time of death, even in human tissues [27,32,36,43,45,47]. In fact, it is known that among the various classes of RNA, mRNA seems to be the most recommendable for this purpose, especially in the case of short time periods, up to 5–7 days [28,39,62,64].

Odriozola A. [27] documented the usefulness of miR-34c, miR-484, miR142-5p, and miR-541 already within the first 24 h after death, and Singh P. [47] within 196 h, proposing a comparative analysis between degradation times of miR-195, miR-206, and miR-378. Both studies were performed on humans. Two other studies conducted on dead human tissues made it possible to evaluate the usefulness of miRNAs in estimating the “long” PMI [46]. Na J. [46] detected a significant decrease in Let-7e and miR-16 in the patella of 71 cadavers, considered statistically significant up to 30 days. Alshehhi S. et al. [45] identified a progressive decrease of miR10b on human semen up to 14 days, while miR-891a and miR-205 (studied on human saliva) remained stable up to 360 days.

It has been said that miRNAs behavior and stability can vary depending on the tissue in which they are analyzed. Currently, the heart, brain, and skeletal muscle appear to be the best tissues on which to perform these studies [28,29,30,36,38,39,40,43,44]. Conversely, a study conducted on venous blood from dead humans produced negative results; therefore, it is currently not considered a good matrix on which to carry out this investigation [32].

Despite the promising results from the little research currently published in the literature, the analysis of miRNAs as a potential new tool for estimating PMI appears to be very hopeful. What emerges is the importance of producing multi-parametric mathematical models that allow the simultaneous integration of information on the behavior of different RNAs, not exclusively miRNAs but also mRNAs.

### 4.2. Limitations

In the end, it must be highlighted that, despite the data already published in the literature, several constraints persist, rendering these investigations still imprecise and challenging to replicate. In particular, the few studies present in the literature analyze a very small number of miRNAs, often making use of variable methods and time intervals. Furthermore, all these studies are burdened by numerous confounding factors, which can reduce their accuracy by altering the concentrations of microRNAs. Among these are the matrix on which the analysis is performed, both the environmental temperatures and those at which the sample is stored, the autolysis and putrefaction phenomena, the pH values, the “agonal state”, and even the analyses that are used to process data. In this regard, only a small number of studies provide data regarding the error rate and/or make use of “mathematical models”, which are fundamental to further reducing the error rate.

## 5. Conclusions

In recent years, there has been a significant growth in forensic research focused on developing new instruments to get a more precise estimate of the post-mortem interval, which is critical in the field of forensic pathology. In this regard, significant attention has been paid to microRNA degradation times. Although the most promising results appear to be obtained using multi-parametric models, the lack of defined and universally acknowledged techniques makes these analyses questionable.

Furthermore, the degradation times of a specific miRNA are influenced by several parameters, including the environmental conditions in which the sample is stored, intrinsic tissue features, and the amount of ribonuclease present in the investigated matrix. Currently, skeletal muscle, the heart, and the brain appear to be the best candidates for these investigations.

Given these limitations, to reach the most accurate results, RNA degradation on several matrices should be examined concurrently. Anyway, considering the first results published in the literature on a terrain yet to be explored, microRNA degradation analyses could represent a potential new tool for achieving a more precise PMI assessment.

## Figures and Tables

**Table 1:** Main data contained in articles with a PMI time frame assessed within 72 h. N.R. *: not reported; N.U. *: not used. N.S.S. *: not statistically significant.

Authors	Year	mRNA	Sample	Tissue	Sample Number	Temperature	PMI—Time Frame Assessed	PMI Significance	PMI Epicrisis	Reference—Control Genes/RNA/DNA—No	Statistical Analysis	Estimated Error
Odriozola A. [27]	2013	miR-34c	Dead human	Vitreous humor	34	N.R. *	Up to 24 h	at least up to 24 h	decrease as PMI increase	miR-222	Two-tailed Student’s *t* test	N.R. *
		miR-222							stable up to 24 h			
		miR-888										
		miR-484							downregulated			
		miR-142-5p							upregulated (* if death occured during night)			
		miR-541										
Sharma et al. [28]	2015	miR-2909	Mice	Heart, lungs, brain, spleen, liver, pancreas and kidneys	9	25 °C	12 h, 24 h, 36 h, 48 h, 72 h	up to 48 h	stable up to 48 if sacrificed at 8:00 pm; stable up to 12 h if sacrificed at 12:00 p.m.	N.U. *	SPSS window v.19 and ANOVA	N.R. *
Lu Y.H. [29]	2016	miR-9 and miR-125b	Rat	Brain	222	5, 15, 25, 35 °C	0, 1, 2, 4, 6, 8, 10, 12, 24 h	miRNA-9 and miRNA-125b remain stable up to 24	stable up to 24 h	5S rRNA, miR-9, miR-125b	Regression analysis by SPSS software 8 (v.19)	Average error rate: 14.1% (β-actin) and 22.2% (GAPDH)
Lu Y.H. [30]	2016	miR-9 and miR-125b	Dead human	Brain	12	N.R. *	from 4.3 to 22.5 h	miRNA-9, miRNA-125b suitable as internal reference markers	stable up to 24 h	5S rRNA, miR-9, miR-125b	Quadratic regression (R software v.19)	Error rate: 24.6% (β-actin) and 41.0% (GAPDH)
Ibrahim S.F. [31]	2019	miR-205 miR-21	Rats	Skin	18	N.R. *	0, 24, 48 h	N.S.S. *	marked increase at 24 h and strong decrease at 48 h	N.R. *	Pearson correlation test	N.R. *
Han L. et al. [32]	2020	miR-3185	Dead human	Heart	51	from 5 °C to 40 °C	from 1.5 h to 30.5 h	N.S.S. *	upregulated in mechanical asphyxia death compared with other death	β-actin mRNA	*t*-test; Mann-Whitney tests	N.R. *
Martinez-Rivera V. [33]	2021	miR-144-3p;	Rats	Skeletal muscle	25	25 °C	0, 3, 6, 12, 24 h	N.S.S. *	decreased at 0–6 h	* To normalize the expression of EPC1: ACTB gene (Rn00667869_m1)	Non-parametric Kruskal Wallis; Mann U Whitney test; Pearson’s Chi-squared test; Cochran-Armitage test; Spearman Rho	N.R. *
		miR-23b-3p;						24 h	significantly down-regulated at 3 to 24 h			
		miR-381-3p						0, 3, 12, 24 h	significantly down-regulated in the first 3 h and upregulated at 6 to 24 h			
Derakhshanfar A. [34]	2022	miR-122	Rats	Liver and kidney	10	25 °C	2, 4, 6, 8, 10, 12, 24, 48 h	N.S.S. *	upregulated at 4, 10, and 24 h; downregulated at 6, 8, and 48 h	miR-16, miR 221, Let-7a, U6 snRNA	Graph Pad Prism8.0 and SPSS 17.0 software	N.R. *
Guardado-Estrada M. [35]	2023	1218 miRNAs, of whom156 downregulated (84) or upregulated (72) at 24 h	Rats	Skeletal muscle	9	22 °C	0, 24 h	N.R. *	miR-139-5p most significant downregulated, rno-miR-92b-5p most significant upregulated	Affymetrix Transcriptome Analysis Console (TAC) SoftwareTM 4.0	Robust Multi-chip Analysis (RMA)	N.R. *

**Table 2:** Main data contained in articles with a PMI time frame assessed within 7 days. N.R. *: not reported. N.S.S. *: not statistically significant.

Authors	Year	mRNA	Sample	Tissue	Sample Number	Temperature	PMI—Time Frame Assessed	PMI Significance	PMI Epicrisis	Reference–Control Genes/RNA/DNA—No	Statistical Analysis	Estimated Error
Wen-Can L. [36]	2014	miR-1, miR-143, miR-208, 18S rRNA	Rats (Sprague-Dawley)	Heart	6	25 °C	1, 3, 6, 12, 15, 18, 21, 24, 36, 48, 72, 96, 120, 144, and 168 h, up to 7 days	up to 168 h	miR-1 has a stable expression along the studied PMI; 18S rRNA gradually increased in the early stage and peaked at around 96 h	miR-1	Variance and linear regression	N.R. *
Pan H. [37]	2014	miR-203	Rat	Skin	18	4, 15, 35 °C	From 0 to 120 h	N.R. *	remain stable up to 120 h	miR-203	Regression analysis (GraphPad)	N.R. *
Ma J. [38]	2015	miR-9, miR-125b (Other RNAs: β-actin, GAPDH and RPS29 mRNA, 5S and 18S rRNA, U6 snRNA and Let-7a)	Rats (Sprague-Dawley)	Brain	270	4, 15, 25, 35 °C	1, 3, 6, 12, 24, 36, 48, 72, 96, 120, and 144 h	N.R. *	miR-9, miR-125b remain stable up to up to 144 days	miR-9, miR-125b	Bivariate cubic curve and quadratic regression	Mean error rate 4.8% Ranging from 30% (30 h at 20 °C) to 43% (10 h at 30 °C)
					36	10, 20, 30 °C	10, 30, 50, 100 h					
Lv Y. [39]	2016	miR-195, miR-200c (lung); miR-1, miR-206, (skeletal muscle)	Rats	Lungs and skeletal muscle	216	10, 20, 30 °C	0, 1, 3, 6, 12, 24, 36, 48, 72, 96, 120, 144 h	Up to 144 h	Stable up to 144 h, then decreased	b-actin and GAPDH, 18S rRNA, RPS29 mRNA	Linear, quadratic, and cubic regression, △Ct method	7.4%
					15	10, 15, 20, 25, 30 °C	10, 60, 110 h					
			Dead human									12.5%
					12	ambient temperature of the crime scene before being transferred to freezer or autopsy	ariable from 7 to 73 h					
Lv Y. [40]	2017	miR-1, miR-133a, miR-122, miR-9, miR-125b	Dead human; rats Sprague-Dawley	Human heart (Apex Cordis), liver (right lobe), and brain (frontal cortex)	Thirteen dead human (seven males, six females); 36 rats	4 °C, 15 °C, 25 °C, 35 °C	From 6–71 h up to 5 days	Up to 71 h	Stable up to 5 days	β-actin, GAPDH, RPS29 mRNA	Linear, quadratic, and cubic regression (R software v. 2.3), ΔCt method	Mean estimated error: 5.06 (human); 2.89 (rat)
Kim S-Y. [41]	2021	miR-16, miR-208b, let-7e, miR-1	Dead human	Venous blood from three different sites (external iliac vein, inferior vena cava, and coronary sinus)	28 (17 male, 11 female)	N.R. *	From 16 to 86 h	N.S.S. *	Variable in relation to the sampling site	Ce_miR-39_1	ΔCt method, Spearson’s correlation, and Tukey’s analysis	N.R. *

**Table 3:** Main data contained in articles with a PMI time frame assessed over 7 days. N.R. *: not reported.

Authors	Year	mRNA	Sample	Tissue	Sample Number	Temperature	PMI—Time Frame Assessed	PMI Significance	PMI Epicrisis	Reference—Control Genes/RNA/DNA—No	Statistical Analysis	Estimated Error
Lv. Y. [42]	2014	miR-125b and miR-143 (Other RNAs: β-actin, GAPDH1 and 2 mRNA, ACTB1 and 2,18S rRNA, U6 snRNA)	Rats (18)	Spleen	6	4, 25 °C	0, 1, 3, 6, 12, 24, 36, 48, 72, 96, 120, and 144 h	At 25 °C, miR-125b and miR-143 stable up to 44 h; at 4 °C, miR-125b and miR-143 stable up to 312 h	miR-125b Ct values fluctuated slightly within 36 h and then increased slowly at 25 °C; the same trend has been observed within 144 h at 4 °C	miR-125b and miR-143 for GAPDH1 and 2, ACTB1 and 2, U6, and 18S rRNA	Linear, quadratic, cubic, reciprocal, exponential, and logarithmic regression (GraphPad v5.0)	<10% at 25 °C and <20% at 4 °C
					6		0, 12, 24, 36, 48, 72, 96, 120, 144, 168, 192, 216, 240, 264, 288, and 312 h					
					3		0, 5, 55 and 105 h					
					3		0, 20, 100, 180, and 260 h					
Tu C. [43]	2018	miR-122, miR133a (and Gapdh, Rps18, β-actin, 5S, 18S, U6, circ-AFF1, LC-Ogdh, LC-LRP6)	Mice BALB/cc	Liver, heart, and skeletal muscle	45	25 °C	0, 1, 2, 3, 4, 5, 6, 7, 8 d	Up to 7.5 days	Stable up to 8 days	geNorm and NormFinder	△Ct method	N.R. *
Tu C. [44]	2019	miR-122, miR133a	Mice BALB/cc	Liver, heart, and skeletal muscle	15	25 °C	0, 1.5, 3.5, 5.5, 7.5 days	From 0 to 6 days	Stable up to 7.5 days	geNorm and NormFinder	Linear, quadratic, and cubic regression	0.62; 0.5 d if the data of all three tissues studied are combined together
Alshehhi S. [45]	2019	miR205	Living human	Fresh saliva	19	Room temperature	0, 7, 14, 28, 90, 180, 270, 360 days	N.R. *	Stable up to 360 days;	ACTB, 18S, U6	Relative expression ratio	N.R. *
		miR10b						Up to 14 days	degrades slightly up to 14 days;			
				Fresh ejaculated semen								
		miR891a						N.R. *	remains stable up to 360 days			
Na J.Y. [46]	2020	let-7e, miR-16	Dead human	Patella	71	N.R. *	<1 month, 1 month, 3 month, 6 month, >6 month	Up to 1 month	Rapid decrease in the first month	Ce_miR-39_1	ΔCt method, unpaired *t*-test	N.R. *
Singh P. [47]	2023	miRNA-195, miRNA-206, miRNA-378	Dead human	Heart (left ventricle)	20	Room temperature	12, 24, 48, 72, 96, 120, 168 and 196 h	From 24 to 196 h	Decrease from 24 to 196 h	miRNA-1	Unpaired *t*-test	N.R. *

## Data Availability

All the data are reported in the paper.

## Supplementary Materials

The following supporting information can be downloaded at: https://www.mdpi.com/article/10.3390/ijms25179207/s1. Reference [65] are cited in the supplementary materials.
